# Alterations in urinary microbiota composition and function in men with Asthenozoospermia: a case-control study

**DOI:** 10.1186/s12610-026-00326-0

**Published:** 2026-10-14

**Authors:** Yang Pan, Shangren Wang, Tingshuai Cao, Bing Li, Xuexue Hao, Muwei Li, Li Liu, Xiaoqiang Liu

**Affiliations:** 1 Department of Gastrointestinal Surgery, The First Affiliated Hospital of Chongqing Medical University, No.1 Youyi Road, Yuzhong District, Chongqing, 400016 China; 2 Department of Urology, Tianjin Medical University General Hospital, Tianjin, China; 3 Department of Minimally Invasive Esophageal Surgery, Tianjin Medical University Cancer Institute & Hospital, Tianjin, China; 4 Department of Urology, Central Hospital Affiliated to Shandong First Medical University, Jinan, China

**Keywords:** Male infertility, Asthenozoospermia, Urinary microbiota, 16S rRNA gene sequencing, Metabolic pathway, Infertilité masculine, Asthénozoospermie, Microbiote urinaire, Séquençage du gène 16S rRNA, Voie métabolique

## Abstract

**Background:**

Asthenozoospermia (AZS) is a leading cause of male-factor infertility, yet the contribution of the urinary microbiota to sperm motility remains poorly defined, because previous work has focused almost exclusively on the seminal and gut microbiota. Here we used 16S rRNA gene sequencing to characterize the composition and functional profiles of the urinary microbiota in men with AZS and in fertile controls.

**Results:**

Isolated AZS and age-matched fertile controls were enrolled, and urinary specimens from participants were sequenced successfully. Alpha diversity of the urinary microbiota did not differ between the two groups. In contrast, beta diversity analysis revealed a statistically significant difference in overall community structure, although the group effect was modest in magnitude (PERMANOVA on Bray-Curtis dissimilarities, R^2^ = 0.13, *p* = 0.001). Eighteen genera differed significantly in relative abundance. *Porphyromonas, Corynebacterium*, and *Finegoldia* were more abundant in men with AZS, whereas *Blautia* and *Bacteroides* were more abundant in controls. Predicted functional analysis identified multiple metabolic and genetic-information-processing pathways that differed between the groups, including replication and repair modules, peptidoglycan biosynthesis, one-carbon pool by folate, and nicotinate and nicotinamide metabolism.

**Conclusions:**

It appears that men with AZS exhibit altered urinary microbiota structure and predicted functional profile. These associations extend the microbiome-male-infertility axis beyond the seminal and gut compartments and may inform biomarker discovery and microbiome-modulating strategies, although longitudinal and mechanistic studies are required to establish causality.

## Résumé

**Contexte:**

L’asthénozoospermie (AZS) est une cause majeure d’infertilité due aux facteurs masculins ; pourtant la contribution du microbiote urinaire à l’altération de la mobilité des spermatozoïdes reste mal définie, car les travaux précédents se sont presque exclusivement concentrés sur le microbiote séminal et digestif. Nous avons utilisé le séquençage du gène 16S rRNA pour caractériser la composition et les profils fonctionnels du microbiote urinaire chez des hommes atteints d’AZS et chez des témoins fertiles.

**Résultats:**

Des hommes présentant une AZS isolée et des témoins fertiles appariés sur l’âge ont été recrutés, et des échantillons urinaires de tous les participants ont été séquencés avec succès. La diversité alpha du microbiote urinaire entre les deux groupes ne différait pour aucun des indices examinés. En revanche, l’analyse de la diversité bêta a révélé une différence significative dans la structure globale de la communauté, bien que l’effet de groupe ait été modeste en magnitude (PERMANOVA sur les différences de Bray-Curtis, R²=0,13 ; p=0,001). Dix-huit genres différaient significativement en abondance relative. Porphyromonas, Corynebacterium et Finegoldia étaient plus riches chez les hommes atteints d’AZS, tandis que Blautia et Bacteroides étaient plus abondants chez les témoins. L’analyse fonctionnelle a identifié de multiples voies métaboliques et de traitement de l’information génétique qui différaient entre les groupes, notamment les modules de réplication et de réparation, la biosynthèse des peptidoglycanes, un réservoir de carbone par folate, ainsi que le métabolisme du nicotinate et de la nicotinamide.

**Conclusions:**

Les hommes atteints d’AZS présentent une structure de microbiote urinaire distincte et un profil fonctionnel altéré. Ces associations étendent l’axe microbiome-infertilité masculine au-delà des compartiments séminal et digestif, et peuvent influencer la découverte de biomarqueurs et les stratégies de modulation du microbiome ; mais des études longitudinales et mécanistiques sont nécessaires pour en établir la causalité.

## Introduction

Asthenozoospermia (AZS) is a principal cause of male-factor infertility and is defined by a marked reduction in sperm motility. According to the edition of the World Health Organization (WHO) laboratory manual, the diagnosis is established when progressive motility falls below the reference value [1]. As one of the most frequently encountered semen abnormalities, AZS accounts for a substantial proportion of infertility worldwide and imposes a considerable burden on reproductive health and on the psychological well-being of couples of childbearing ages [2, 3].

The pathophysiology of AZS is multifactorial and encompasses genetic variation, structural defects of the sperm flagellum, and mitochondrial dysfunction. Among the mechanisms proposed, excessive generation of reactive oxygen species (ROS) and the resulting oxidative stress are regarded as pivotal, because they compromise both the kinetic energy and the membrane integrity of spermatozoa [4]. Despite the high clinical prevalence of the condition, the mechanisms that drive the decline in motility remain only partially resolved, which underscores the need to examine the wider biological and microenvironmental determinants of male reproductive function [5].

High-throughput sequencing has established that the urinary tract, long regarded as sterile, harbors a diverse microbial community that contributes to urogenital homeostasis [6, 7]. Imbalance within this community has been implicated in several urologic disorders, including urinary tract infection [8], urinary incontinence [9], and genitourinary malignancy [10]. Longitudinal multi-omics work has further shown that dysbiosis of the gut reservoir precedes and predicts recurrent urinary tract infection, underscoring the existence of a gut-bladder axis [11], and the urinary compartment has consequently emerged as an accessible, non-invasive window onto urogenital host-microbe interaction [12]. In men, the urinary and seminal tracts share the urethral passage, so the urinary community may plausibly report on inflammatory processes within the urogenital tract that bear on sperm function. Whether such a signature exists in men with impaired motility has not been established, and defining it may clarify the microenvironmental contributors to AZS [13].

Despite accumulating evidence that the microbiome influences male reproductive health, the urinary microbiota of men with AZS remains essentially uncharacterized: most microbiome studies in male infertility have interrogated semen or stool, whereas the urinary microbiotae has received little attention. In the present study, we applied 16S rRNA gene sequencing to profile the urinary microbiota of men with AZS and of fertile controls. Our objectives were to identify differentially abundant taxa and to explore the predicted functional pathways associated with AZS, thereby providing new insight into the potential role of urinary microbiota in sperm motility.

## Men and methods

### Participant recruitment and selection criteria

Men with AZS and fertile controls were recruited through the health examination center and the fertility counseling service of the hospital. The protocol was reviewed and approved by the institutional review board and the study was conducted in accordance with the Declaration of Helsinki. A standardized questionnaire was administered to capture demographic data, lifestyle factors, family history, and occupation; participants were interviewed by trained staff to promote accurate and complete reporting.

To preserve the integrity of the microbiota analysis and to limit confounding by conditions such as infection and by concurrent medication, stringent eligibility criteria were applied. The AZS group comprised men aged 18 to 40 years with isolated AZS, defined according to the edition of the WHO laboratory manual [1] as progressive motility below reference value in the presence of an ejaculate volume, sperm concentration, total sperm count, and proportion of morphologically normal forms within the reference ranges. The diagnosis was confirmed on semen analyses, and only men who met the criteria were enrolled. Men with oligozoospermia, teratozoospermia, azoospermia, or any combined semen abnormality were ineligible.

The control group comprised fertile volunteers with normozoospermia on semen analyses, each of whom had fathered at least one child within the preceding three years through natural conception. Controls were frequency-matched to the AZS group by age in two-year strata. The following exclusion criteria were applied to both groups: (i) use of systemic antibiotics, probiotics, or immunosuppressive agents within two months before sampling; (ii) active urinary or genital tract infection, leukocytospermia, clinically detectable varicocele, febrile illness within the preceding three months, or any systemic inflammatory condition; and (iii) other conditions affecting sperm quality or microbiota characteristics, such as a history of malignancy, autoimmune disease, diabetes mellitus, or chronic kidney disease.

### Semen analysis

All participants observed a standardized abstinence interval of 2 to 7 days before sampling, and recent sexual activity was documented in the questionnaire to account for its known transient effect on the urogenital microbiota. Abstinence duration did not differ between the groups (Table 1). Semen parameters were assessed according to the edition of the WHO laboratory manual for the examination and processing of human semen [1]. After complete liquefaction at 37 °C, ejaculate volume was determined by weighing. Sperm concentration and motility were evaluated using a computer-assisted sperm analysis (CASA) system: a 10 µL aliquot of well-mixed semen was loaded into a pre-warmed (37 °C) Makler counting chamber, and at least 200 spermatozoa in a minimum of five randomly selected fields were assessed for each specimen. Progressive motility was defined as the sum of rapid and slow progressive spermatozoa. Sperm morphology was evaluated independently on Diff-Quik-stained smears by two experienced technicians blinded to group allocation, with at least 200 spermatozoa classified per slide according to the WHO criteria; discrepant slides were re-read and adjudicated by consensus. All analyses were performed by the same laboratory using the same instrument settings throughout the study period.

**Table 1:** Demographic, lifestyle, and semen characteristics of asthenozoospermia patients and fertile controls

	AZS (n = 60)	Control (n = 60)	*P***-value**
Age (years)^a^	28.3 (3.1)	28.3 (2.9)	0.927
Body mass index (kg/m^2^)^a^	25.1 (4.1)	24.8 (3.7)	0.672
Smoking status, n (%)^c^			0.841
*No*	43 (71.7%)	42 (70%)	
*Yes*	17 (28.3%)	18 (30%)	
Drinking status, n (%)^c^			0.459
*No*	37 (61.7%)	33 (55%)	
*Yes*	23 (38.3%)	27 (45%)	
Abstinence Time, days^b^	4.0 (2.3, 5)	4.0 (3, 5)	0.317
Semen Volume, mL^b^	3.7 (2.7, 4.7)	3.6 (3.0, 4.4)	0.981
Sperm Concentration, 10^6^/mL^b^	54.0 (29.4, 95.0)	68.6 (37.2, 108)	0.127
Progressive Motility (%)^b^	22.5 (12.9, 26.3)	45.5 (40.7, 56.1)	<0.001
Normal Morphology (%)^b^	5.3 (4.2, 6.9)	5.8 (4.5, 7.6)	0.416

^a^ Normally distributed continuous variables are presented as mean (standard deviation) and were compared with Student’s t test

^b^ variables departing from normality are presented as median with interquartile range and were compared with the Mann-Whitney U test

^c^ Categorical variables are presented as counts with percentages and were compared with the chi-square test

AZS, asthenozoospermia

### Urine sample collection and processing

Clean-catch midstream urine was collected using a standardized sterile technique designed to minimize contamination from the external genitalia. The midstream fraction was chosen because it yields a reproducible specimen that limits the contribution of distal urethral and external genital colonizers and maximizes comparability with the established bladder and urinary microbiome literature. Given the low microbial biomass of urine, an optimized protocol was used to maximize DNA yield. Approximately 50 mL of urine was obtained from each participant and transported to the laboratory on ice without delay. The microbial fraction was concentrated by centrifugation at 10,000 g for 20 min at 4 °C; the supernatant was discarded and the pellet was resuspended in phosphate-buffered saline (PBS) for DNA extraction.

DNA was extracted from the resuspended pellets with the QIAamp DNA Mini Kit (Qiagen, Germany) according to the manufacturer’s instructions, with minor modifications to improve lysis of Gram-positive organisms. DNA quantity and integrity were assessed by NanoDrop spectrophotometry (Thermo Fisher Scientific, USA) and agarose gel electrophoresis. To distinguish genuine biological signal from reagent or laboratory contamination, extraction blanks (reagents without urine) and PCR no-template controls were processed alongside the biological specimens throughout the entire workflow.

### 16S rRNA gene sequencing and data analysis

Total bacterial load was quantified by real-time quantitative PCR (qPCR) with universal bacterial primers targeting the 16S rRNA gene, in order to determine whether microbial abundance differed between the groups. For library preparation, the V3–V4 region of the 16S rRNA gene was amplified with primers 341F (5’-CCTACGGGNGGCWGCAG-3’) and 806 R (5’-GGACTACHVGGGTWTCTAAT-3’). Cycling conditions comprised an initial denaturation at 98 °C for 30 s, 30 cycles of 98 °C for 10 s, 55 °C for 30 s, and 72 °C for 30 s, and a final extension at 72 °C for 5 min. Amplicons were purified with Agencourt AMPure XP beads (Beckman Coulter, USA) and quantified on a Qubit fluorometer (Thermo Fisher Scientific, USA). Specimens that failed to amplify or that yielded DNA concentrations below the limit of detection were excluded from downstream analysis.

Sequencing was performed on the Illumina MiSeq platform with paired-end reads. Raw data were processed in Quantitative Insights Into Microbial Ecology 2 (QIIME2) for quality control and taxonomic assignment [14]. Reads were demultiplexed and primers were trimmed. Quality filtering, error correction, chimera removal, and inference of amplicon sequence variants (ASVs) were performed with the DADA2 pipeline. To address contamination rigorously, the R package decontam (version 1.12.0) was applied using the prevalence method [15]; ASVs that were more prevalent in negative controls than in biological samples, together with recognized laboratory contaminants, were removed before downstream analysis. ASVs were classified against the SILVA 138 reference database. Sequencing depth was normalized by rarefaction to the library with the lowest verified read count to avoid bias from uneven depth.

Alpha and beta diversity were evaluated to characterize community richness and structure, using the observed features, Chao1, Shannon, and Simpson indices and Bray–Curtis dissimilarity. Principal coordinate analysis (PCoA) was performed with the vegan package in R. Differences in community structure were tested by permutational multivariate analysis of variance (PERMANOVA) on Bray-Curtis dissimilarities, and homogeneity of multivariate dispersion was verified by PERMDISP to confirm that the observed separation did not merely reflect unequal within-group dispersion. Linear discriminant analysis (LDA) effect size (LEfSe) was used to identify differentially abundant taxa (*p* < 0.05 and LDA score > 3.0) [16]. Functional potential was inferred from the ASV table with Phylogenetic Investigation of Communities by Reconstruction of Unobserved States 2 (PICRUSt2) [17].

### Statistical analyses

Analyses were performed in SPSS (version 23.0) and R (version 4.1.3). Normality of continuous variables was assessed with the Shapiro-Wilk test. Normally distributed variables are presented as mean ± standard deviation and were compared with Student’s t test; variables departing from normality are presented as median with interquartile range and were compared with the Mann-Whitney U test. Categorical variables are presented as counts with percentages and were compared with the chi-square test. Alpha diversity indices were compared with the Mann-Whitney U test. Differentially abundant taxa identified by LEfSe were considered significant at *p* < 0.05 with an absolute LDA score above 3.0. Differences in PICRUSt2-predicted KEGG pathway abundances were presented as the difference in mean relative abundance with 95% confidence intervals. All tests were two-sided, and *p* < 0.05 was considered statistically significant.

## Results

### General characteristics of AZS patients and fertile controls

**Fig. 1:**
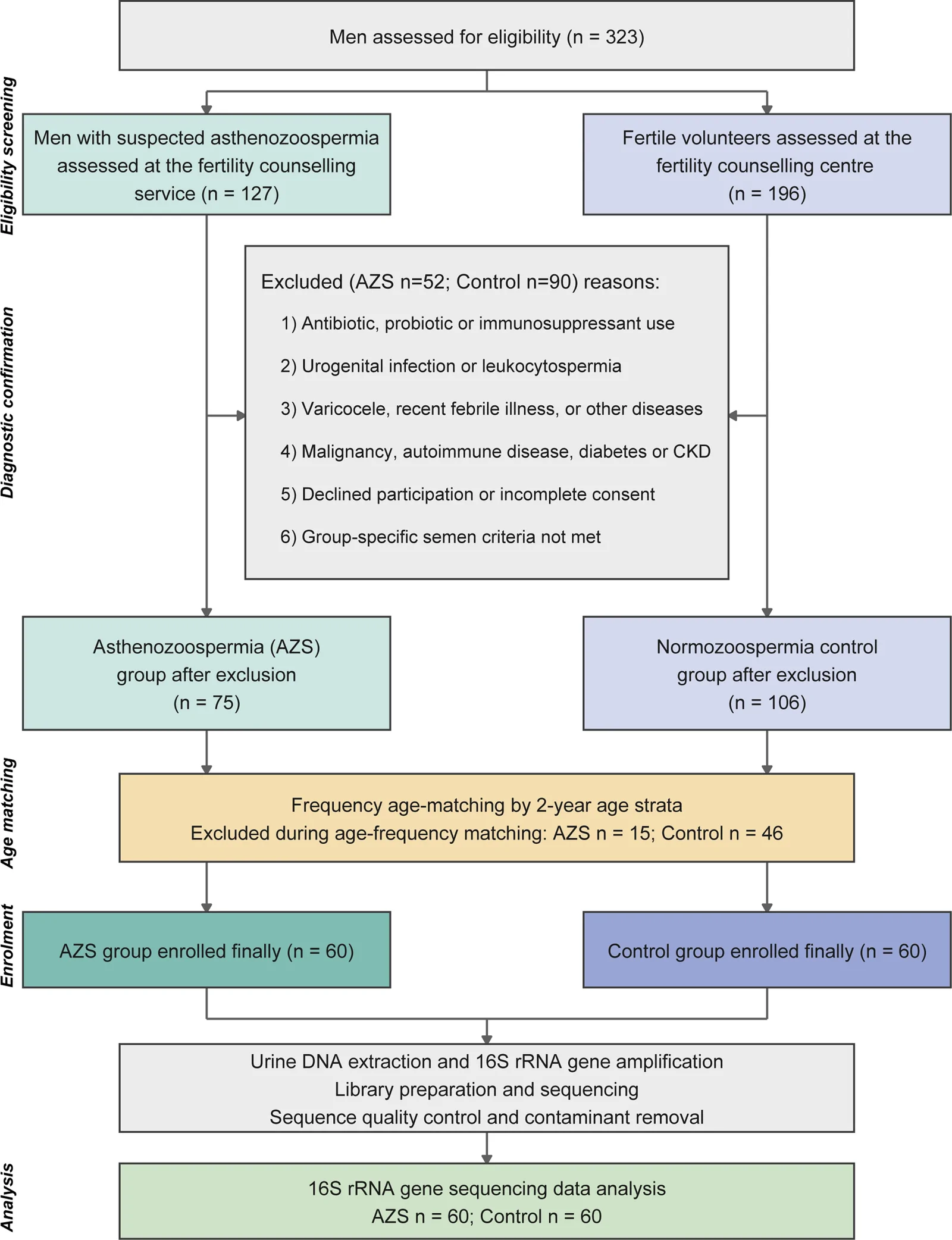
Study flow chart. Flow of participants through assessment, exclusion with reasons, enrollment, and sequencing analysis, presented separately for the asthenozoospermia (AZS) and fertile control groups, together with the final numbers analyzed

The flow of participants through assessment, exclusion, enrollment, and analysis is summarized in Fig. 1. Sixty men with AZS and sixty age-matched fertile controls were enrolled. The 120 specimens that amplified successfully and yielded DNA above the limit of detection were retained for sequencing analysis. Demographic and clinical characteristics of the analyzed participants are summarized in Table 1.

The groups did not differ in age (28.3 ± 3.1 versus 28.3 ± 2.9 years, *p* = 0.927) or body mass index (25.1 ± 4.1 versus 24.8 ± 3.7 kg/m^2^, *p* = 0.672). Lifestyle factors, including smoking and alcohol consumption, were likewise comparable (*p* = 0.841 and *p* = 0.459, respectively). Abstinence duration, ejaculate volume, sperm concentration, and the proportion of morphologically normal spermatozoa did not differ between the groups (*p* > 0.05). As expected from the case definition, progressive motility was lower in the AZS group than in fertile controls (median 22.5% versus 45.5%, *p* < 0.001).

### Diversity of the urinary microbiota

To determine whether sperm motility is accompanied by alterations in the urinary microbiota, we first characterized global community features. Quality filtering and decontamination yielded high-quality sequence data for all libraries. The PCR of the 16S rRNA gene confirmed that the bacterial load of every urine specimen exceeded that of the extraction blanks and no-template controls, and total bacterial load did not differ between the AZS and control groups. Sequencing depth was ample, with Good’s coverage exceeding 99% in libraries, confirming that the majority of phylotypes had been captured.

**Fig. 2:**
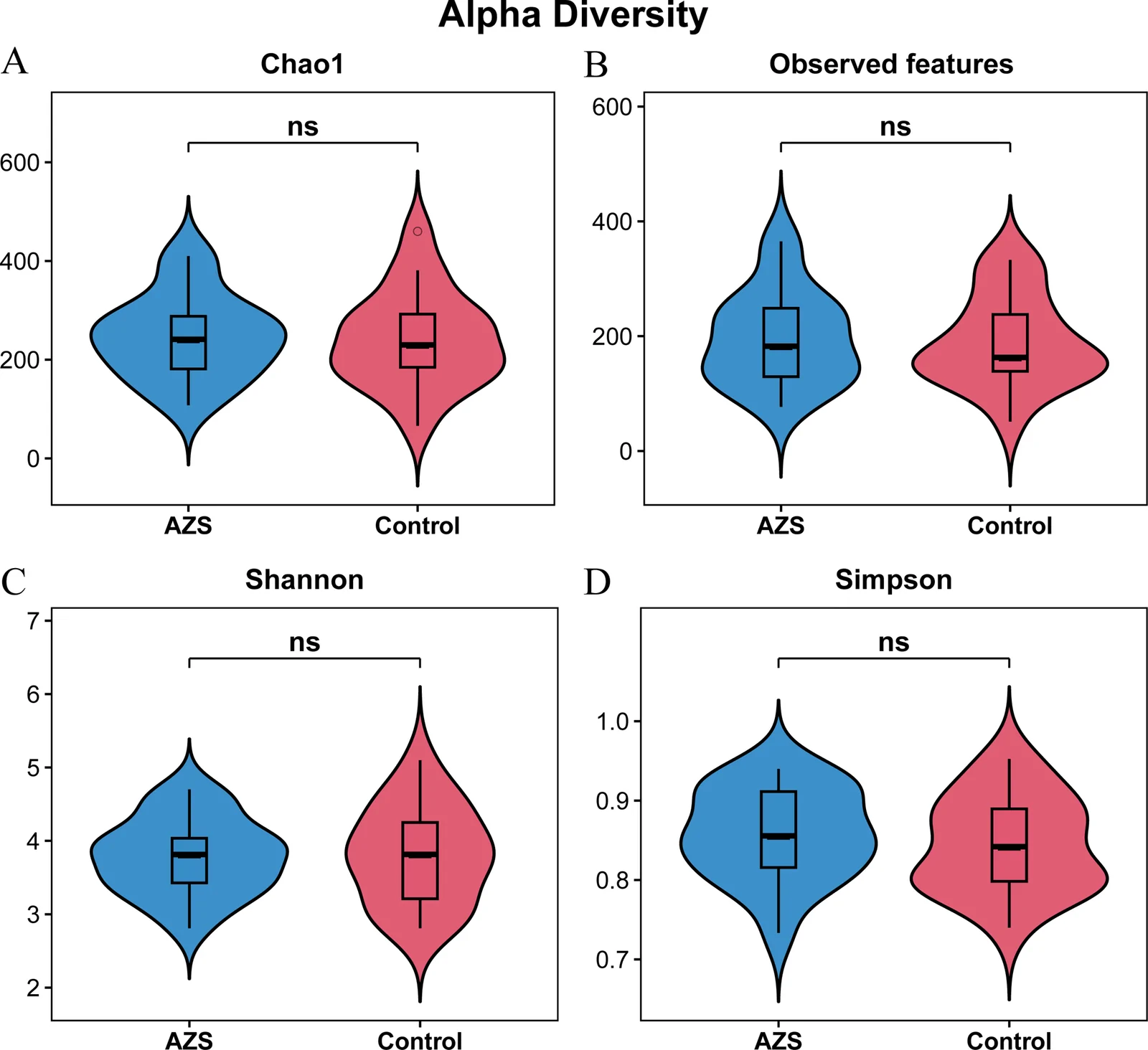
Alpha diversity of the urinary microbiota in men with asthenozoospermia and fertile controls. Alpha diversity was assessed using four indices: (**A**) Chao1 index; (**B**) Observed features; (**C**) Shannon index; (**D**) Simpson index. Between-group comparisons were performed with the Mann-Whitney U test; ns, *p* > 0.05. AZS, asthenozoospermia

**Fig. 3:**
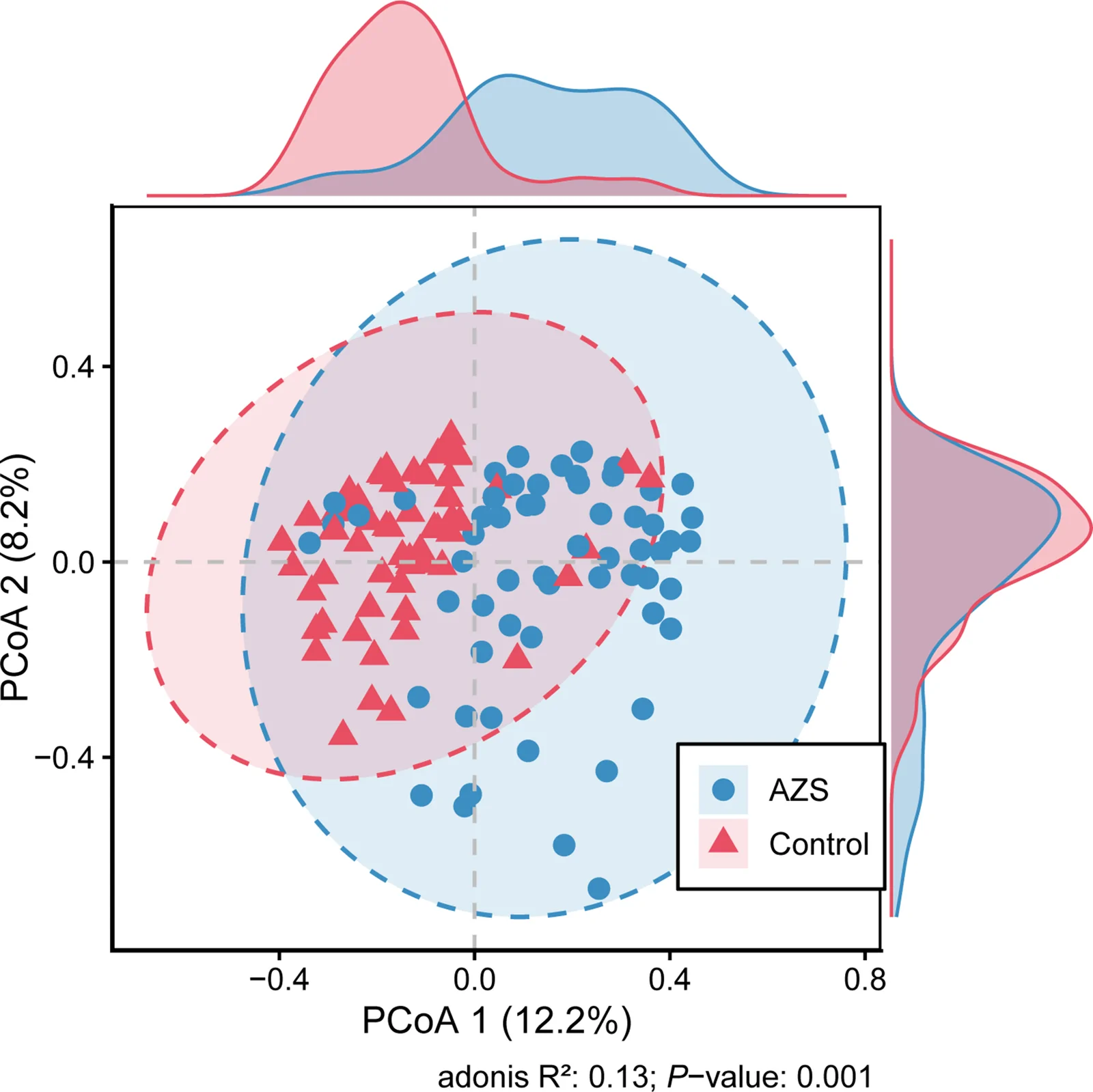
Beta diversity of the urinary microbiota in men with asthenozoospermia and fertile controls. Principal coordinate analysis (PCoA) based on Bray-Curtis dissimilarity revealed partial separation of urinary communities between the two groups. AZS, asthenozoospermia; PCoA, principal coordinate analysis

Alpha diversity did not differ between the groups by any metric examined, including observed species, Chao1, Shannon, and Simpson indices (*p* > 0.05, Fig. 2). In contrast, principal coordinate analysis (PCoA) based on Bray-Curtis dissimilarity showed partial separation of the two groups along the first two coordinates (Fig. 3), and PERMANOVA confirmed that urinary community structure differed significantly between men with AZS and fertile controls (Bray-Curtis dissimilarity, R^2^ = 0.13, *p* = 0.001). PERMDISP showed no significant between-group difference in multivariate dispersion, reducing concern that the PERMANOVA result was driven primarily by unequal within-group dispersion. To assess the robustness of the group effect, PERMANOVA was repeated with body mass index, smoking status, alcohol consumption, and abstinence duration entered as covariates, and the group term remained a significant predictor of community structure. It should nonetheless be emphasized that group membership accounted for only approximately 13% of the total variance in community structure; the remaining variance is presumably attributable to other host-related, behavioral, and environmental determinants. AZS-associated microbiota alteration should therefore be regarded as one modest contributor among many rather than the dominant determinant of the urinary community.

### Taxonomic analysis of urinary microbiota

**Fig. 4:**
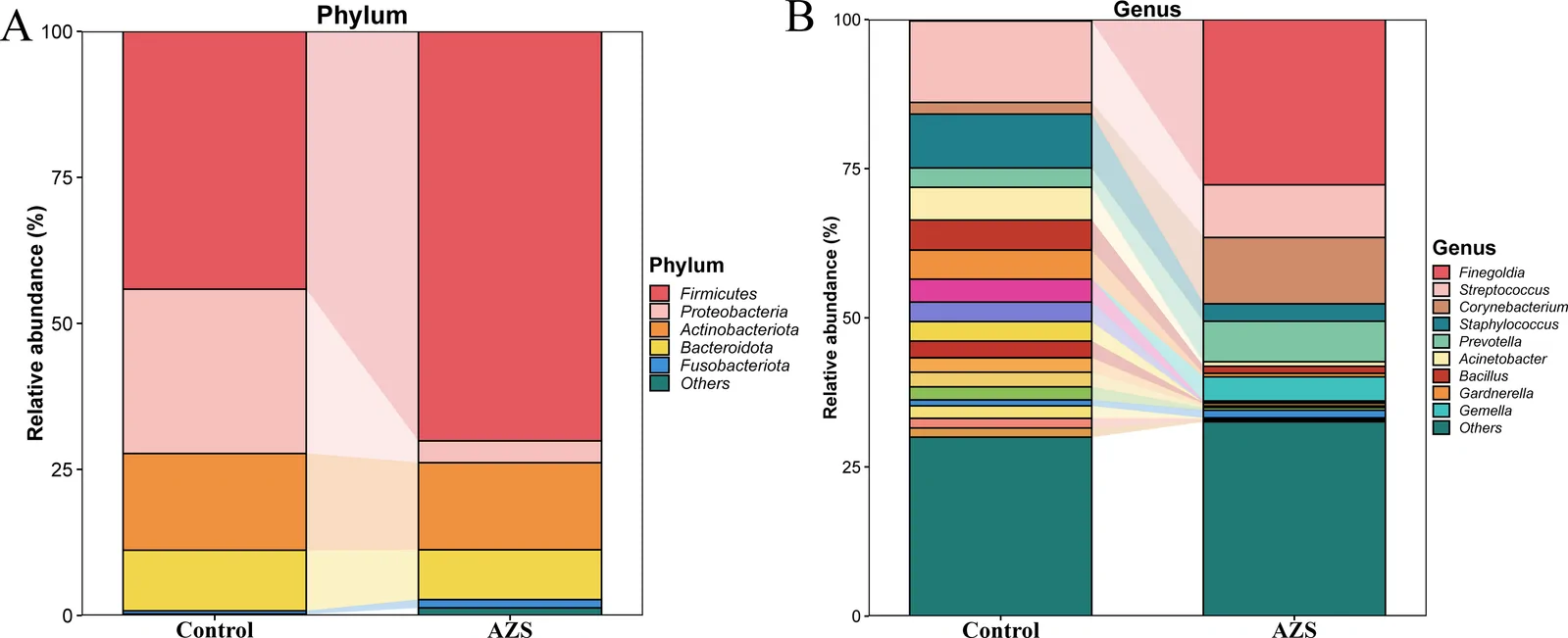
Taxonomic composition of the urinary microbiota in men with asthenozoospermia and fertile controls. (**A**) Relative abundance at the phylum level, showing *Firmicutes, Proteobacteria, Actinobacteriota*, and *Bacteroidota* as the dominant phyla in both groups. (**B**) Relative abundance at the genus level. The five most abundant genera in the AZS group included *Finegoldia, Streptococcus*, *Corynebacterium, Staphylococcus*, and *Prevotella*, whereas *Streptococcus, Staphylococcus*, *Acinetobacter, Bacillus*, and *Gardnerella* predominated in controls. The 20 most abundant genera are shown as individual colored segments in the bars, with all remaining taxa pooled as "Others"; for clarity, only the nine most abundant genera are labeled in the legend. Bars represent mean relative abundances. AZS, asthenozoospermia

We next compared community composition at the phylum and genus levels. At the phylum level, the dominant taxa in both groups were *Firmicutes*, *Proteobacteria*, *Actinobacteriota*, and *Bacteroidota* (Fig. 4A). At the genus level, the five most abundant genera in men with AZS were *Finegoldia*, *Streptococcus*, *Corynebacterium*, *Staphylococcus*, and *Prevotella* (Fig. 4B), whereas *Streptococcus*, *Staphylococcus*, *Acinetobacter*, *Bacillus*, and *Gardnerella* predominated in fertile controls. Consistent with the beta diversity analysis, these profiles indicate that the composition of the urine community may differ between AZS and fertile controls.

### Key urinary genera associated with AZS

**Fig. 5:**
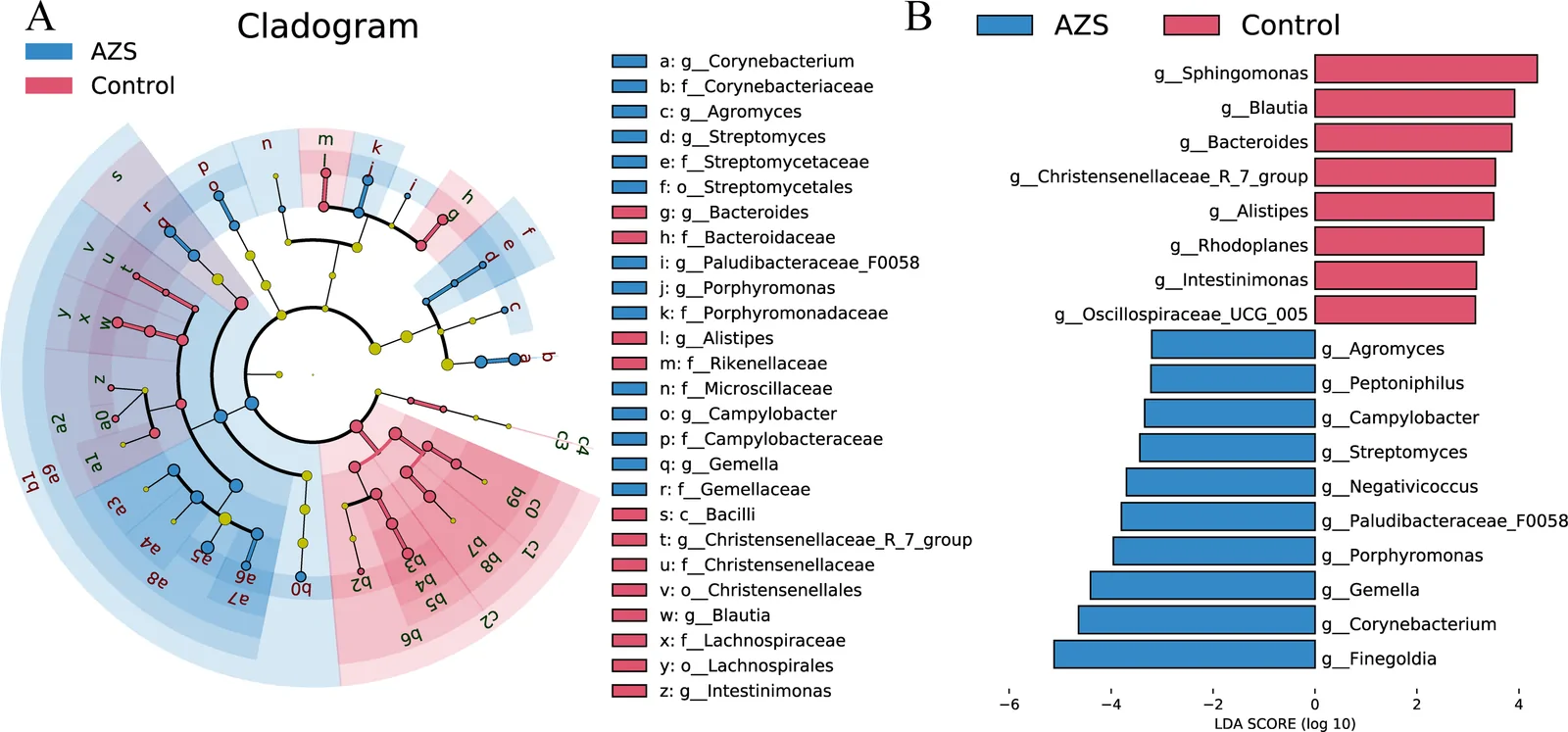
Differentially abundant urinary taxa identified by linear discriminant analysis effect size (LEfSe). (**A**) Cladogram showing taxa enriched in the asthenozoospermia and fertile control groups. For clarity, only taxa labeled a-z are annotated in the legend; the remaining nodes are indicated by abbreviations on the plot. (**B**) Linear discriminant analysis (LDA) scores of differentially abundant genera. Only taxa meeting an LDA score threshold of 3.0 and *p* < 0.05 are shown. AZS, asthenozoospermia; LDA, linear discriminant analysis; LEfSe, linear discriminant analysis effect size

LEfSe was applied to define the taxa that discriminate between the two groups. Fifty-one discriminative features were identified (Fig. 5), among which 18 genera differed significantly in relative abundance. *Blautia*, *Bacteroides*, *Intestinimonas*, *Sphingomonas*, *Alistipes*, *Christensenellaceae R-7 group*, *Rhodoplanes*, and *Oscillospiraceae*_*UCG-005* were more abundant in fertile controls. Conversely, *Porphyromonas*, *Corynebacterium*, *Streptomyces*, *Peptoniphilus*, *Negativicoccus*, *Agromyces*, *Paludibacteraceae_F0058*, *Finegoldia*, *Campylobacter*, and *Gemella* were more abundant in the AZS group.

### Functional analysis of the urinary microbiota

Having established that the urinary community of men with AZS is compositionally altered, we used PICRUSt2 to assess whether these differences translate into altered functional potential, and mapped the inferred gene content to the Kyoto Encyclopedia of Genes and Genomes (KEGG) hierarchy.

**Fig. 6:**
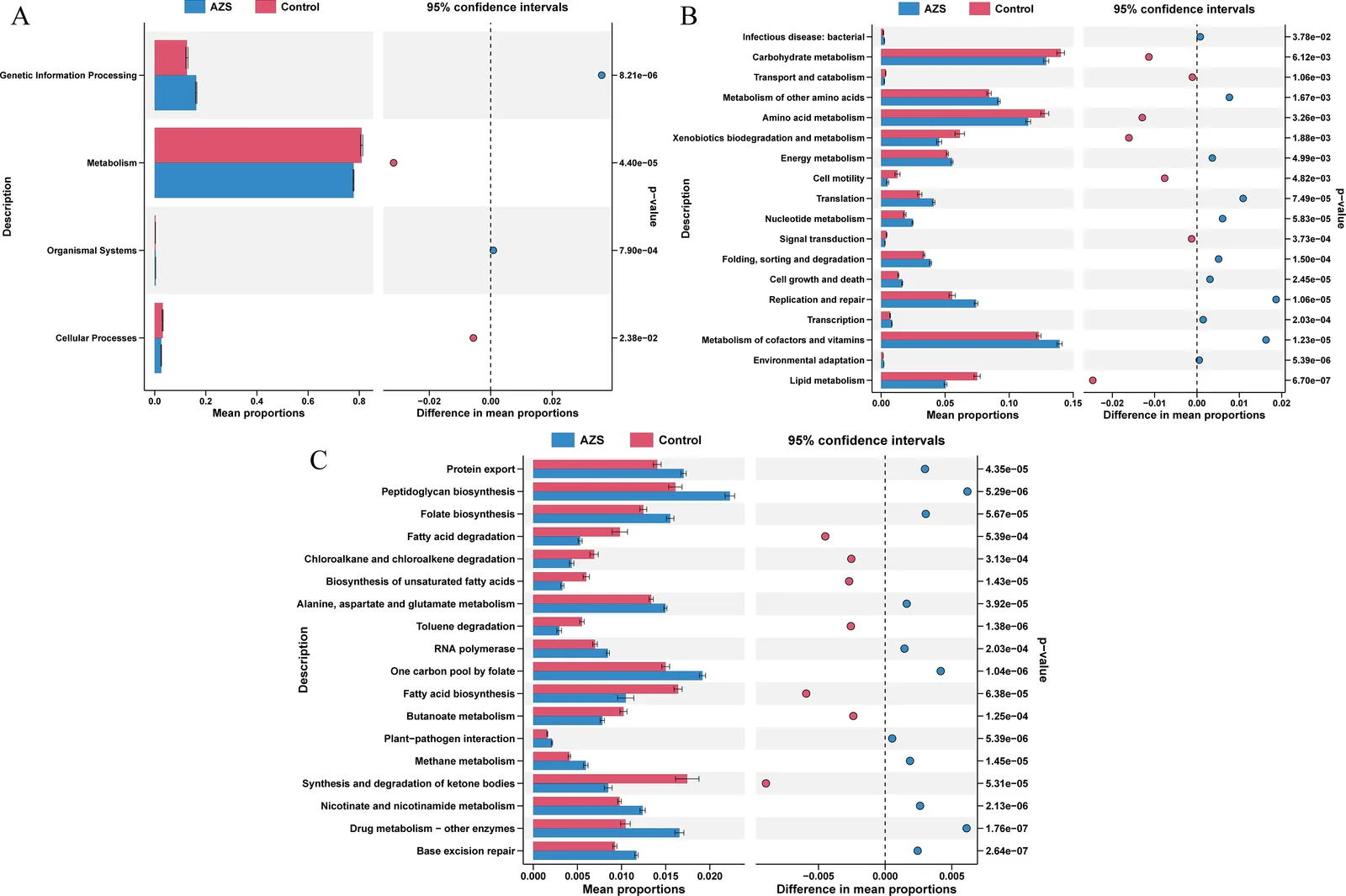
Predicted functional profiles of the urinary microbiota in men with AZS and fertile controls inferred by PICRUSt2. Differentially abundant KEGG pathways at (**A**) level 1, (**B**) level 2, and (**C**) level 3 of the KEGG hierarchy. For level 3, the 18 representative differentially abundant pathways are shown. Mean relative abundances are shown for the AZS group (blue) and the control group (red); differences represent the mean relative abundance in the AZS group minus that in the control group, with 95% confidence intervals. Pathways enriched in the AZS group are plotted on the positive axis. AZS, asthenozoospermia; KEGG, Kyoto Encyclopedia of Genes and Genomes; PICRUSt2, Phylogenetic Investigation of Communities by Reconstruction of Unobserved States 2

Four KEGG level 1 categories, 18 level 2 pathways, and 89 level 3 pathways differed significantly between the groups. At level 1, organismal systems and genetic information processing were enriched in the AZS group, whereas metabolism and cellular processes were relatively enriched in fertile controls (Fig. 6A). At level 2, eleven categories were enriched in men with AZS, comprising cell growth and death, energy metabolism, environmental adaptation, folding, sorting and degradation, bacterial infectious disease, metabolism of cofactors and vitamins, metabolism of other amino acids, nucleotide metabolism, replication and repair, transcription, and translation; the remaining seven differentially abundant level 2 categories were enriched in fertile controls (Fig. 6B). At level 3, a greater number of pathways with between-group differences were enriched in men with AZS, such as drug metabolism by other enzymes, base excision repair, one-carbon pool by folate, nicotinate and nicotinamide metabolism, peptidoglycan biosynthesis, protein export, and folate biosynthesis (Fig. 6C).

## Discussion

This study profiled the urinary microbiota of men with AZS and compared it with that of fertile controls. Urinary community structure differed modestly between the groups, and a defined set of genera showed differential abundance in AZS. Together, these observations indicate that alteration of the urinary microbiota might be associated with AZS and may be relevant to its pathophysiology.

We detected no reduction in alpha diversity in the urine of men with AZS. Although reduced diversity in the gut has frequently been associated with disease [18], the relationship between diversity and urinary tract pathology is inconsistent: reduced bacterial diversity has been reported in interstitial cystitis [19], increased diversity in urgency urinary incontinence [20] and chronic prostatitis [21], and no change in overactive [22] or neuropathic bladder symptomatology [23]. It is therefore plausible that the abundance of particular organisms in urine is more informative than the total number of taxa present, a question that future work could resolve by absolute quantification of individual taxa alongside diversity and composition. In our cohort, beta diversity analysis revealed a modest separation of community structure despite unchanged alpha diversity, indicating that AZS might be associated with reorganization of the urinary community rather than with loss of richness or evenness. This pattern is concordant with several microbiome studies of male infertility and other urogenital disorders, in which disease-associated microbiota dysbiosis might manifest principally in community structure rather than in alpha diversity metrics. The literature is nonetheless heterogeneous, and the evidence linking individual taxa to sperm motility remains limited and at times contradictory. Our findings should accordingly be interpreted as associations rather than causal relationships, and our PICRUSt2-based inferences as predictive hypotheses that require metagenomic and metabolomic confirmation.

Some taxa were more abundant in the urine of men with AZS. *Porphyromonas*, *Corynebacterium*, and *Finegoldia* were more abundant in the AZS group, whereas *Blautia* and *Bacteroides* predominated in fertile control. Some genera showed higher relative abundance in the AZS group, although whether they represent viable organisms or clinically relevant pathogens remains unknown.Notably, several of the AZS-enriched taxa have previously been implicated in some inflammatory conditions of the urogenital tract. *Porphyromonas* has been detected in the urine of men with prostate cancer [24], and increased *Porphyromonas* abundance has been reported in men with bladder cancer relative to controls [25]. *Corynebacterium*, conventionally dismissed as a urine culture contaminant, can cause encrusted cystitis, a form of chronic urinary tract infection accompanied by pain, bladder necrosis, and renal failure [26]. Because *Corynebacterium* is a commensal of the skin, urethra, mucous membranes, and genital tract yet is recoverable from patients with urinary tract infection, it is best regarded as a conditional pathogen [27]. *Streptomyces*, a soil-associated Gram-positive genus reported only occasionally as an opportunistic human pathogen, was also more abundant in AZS; its presence in urine more plausibly reflects environmental exposure than a pathogenic contribution, and this observation requires independent confirmation [28].

Although the presence of these organisms does not establish causality, their enrichment might sustain a pro-inflammatory microenvironment detrimental to sperm motility, and several mechanistically coherent hypotheses can be advanced. First, expansion of Gram-negative anaerobes such as *Porphyromonas* might increase the local burden of lipopolysaccharide (LPS). LPS potentially engages Toll-like receptor 4 (TLR4) on resident leukocytes and germline-adjacent cells, activates NF-κB signaling, and drives release of TNF-α, IL-6, and IL-1β. This cytokine-rich milieu might amplifie ROS production, the mechanism most consistently implicated in AZS, whereby excess ROS may peroxidize the polyunsaturated fatty acid-rich sperm membrane, impair axonemal and mitochondrial function, and deplete the ATP required for flagellar beating, thereby reducing progressive motility. Second, *Porphyromonas* associated species might produce proteolytic virulence factors as well as volatile sulfur compounds and metabolites capable of inhibiting mitochondrial respiration; given the absolute dependence of motility on ATP generated along the flagellar midpiece, these metabolites might offer a potential link between *Porphyromonas* enrichment and impaired sperm motility. Third, *Corynebacterium* and *Finegoldia* have been associated with chronic, frequently subclinical urogenital inflammation, however, whether their urinary enrichment has functional consequences for sperm motility remains unknown. These taxa might perturb epididymal sperm maturation, and alter seminal plasma composition; sustained immune activation within the genitourinary tract may further promote fibrotic remodeling of the reproductive tract [29]. Conversely, relative depletion of short-chain fatty acid (SCFA)-producing commensals such as *Blautia* and *Bacteroides* may reflect an environment less conducive to pro-inflammatory taxa, though the biological significance of SCFA production in urine warrants clarification. Although Blautia and Bacteroides have been implicated in microbial metabolite production, particularly in the gut, their functional significance within the urinary compartment remains unclear. Whether changes in these genera contribute to local inflammatory or metabolic alterations requires direct investigation. Taken together, local inflammation, oxidative stress, and altered microbial metabolite signaling might constitute a biologically plausible axis linking urinary microbiota alteration to sperm motility. We emphasize that these mechanisms are hypotheses derived from associative data and require direct experimental validation.

A further methodological consideration concerns the urinary fraction sampled. The first-void fraction preferentially samples the distal urethra and is often favored in male urogenital studies, whereas midstream urine might be designed to partially represent the bladder and upper urinary tract while minimizing urethral input; a post-prostatic-massage (VB3) specimen further enriches for prostatic taxa [30]. Our use of midstream urine therefore characterizes the broader urinary ecosystem and should not be read as a direct proxy for the seminal, prostatic, or epididymal microenvironment. Although the urinary and seminal microbiota overlap partially by virtue of the shared urethral passage, they are not interchangeable: the urinary compartment may report an inflammatory milieu that influences reproductive function indirectly, through local and systemic inflammatory mediators, rather than through direct seminal colonization. Studies that obtain paired first-void, midstream, VB3, and semen specimens from the same individuals will be required to resolve fraction-specific signals and their differential relevance to motility.

Beyond composition, PICRUSt2-based prediction indicated divergence between the groups in genetic information processing, energy metabolism, and metabolism of cofactors and vitamins. Enrichment in AZS of replication and repair modules, comprising base excision repair, nucleotide metabolism, and mismatch repair, together with the transcription and translation categories, might indicate enrichment of taxa whose genomes are equipped for DNA repair and rapid growth, although PICRUSt2 cannot indicate whether these genes are expressed. In parallel, enrichment of peptidoglycan biosynthesis and of the bacterial infectious disease category might be consistent with expansion of taxa equipped with established colonization and virulence machinery, whereas differences in one-carbon pool by folate and in nicotinate and nicotinamide metabolism may point to a shift in the redox and methyl-donor economy of the community, both of which may bear on the antioxidant capacity of the surrounding microenvironment. We stress that these pathways were inferred from 16S rRNA gene profiles rather than measured directly; PICRUSt2 predictions depend on the availability of reference genomes and cannot resolve strain-level functional variation. They should therefore be regarded as hypothesis-generating only, and shotgun metagenomic, metatranscriptomic, and metabolomic studies are required for validation.

## Limitations of the study

Several limitations warrant acknowledgment. First, the cross-sectional, observational design precludes causal inference; reverse causation, whereby impaired reproductive physiology shapes the microbiota, cannot be excluded. Second, the PERMANOVA effect size indicates that AZS status explains only a modest fraction of community variance, and unmeasured factors including diet, sexual activity, host genetics, and subclinical inflammation are likely to exert greater influence. Third, no consensus gold-standard urinary fraction exists for male infertility microbiome research, and our choice of the midstream fraction may have shaped the taxa recovered relative to first-void or VB3 specimens. Fourth, despite sterile collection, negative controls, and in silico decontamination, low-biomass specimens such as urine may remain inherently vulnerable to residual contamination. Fifth, idiopathic AZS is etiologically heterogeneous, and we did not undertake genetic or ultrastructural sub-phenotyping. Sixth, our functional conclusions rest on PICRUSt2 prediction rather than direct metagenomic sequencing. PICRUSt2 infers the gene content (functional potential) encoded by the community from 16S rRNA gene profiles and does not measure gene expression or metabolic activity; predicted pathway differences therefore reflect shifts in the taxonomic and genomic composition of the community rather than its physiological or stress state. Seventh, although a standardized abstinence interval of 2 to 7 days was required and abstinence duration was comparable between groups, this information was self-reported, so residual confounding by recent sexual or ejaculatory activity, which transiently perturbs the urogenital microbiota, cannot be fully excluded. Finally, the single-center design and modest sample size may limit generalizability. The urinary microbiota described here reflects our specific study population rather than a universal signature, and our design cannot establish that the urinary compartment plays a determinative role in impaired sperm motility. The associations should therefore be viewed as compartment-specific correlates, not as evidence that urinary microbiota outweighs microbial signals from other niches in shaping the AZS phenotype.

## Conclusion

In summary, our findings suggest that men with AZS may harbour an altered urinary microbiota that is structurally and potentially functionally different from that of fertile controls. Notwithstanding the limitations outlined above, these findings may provide new insight into the association between urinary microbiota and sperm motility and extend the microbiome-male infertility axis beyond the seminal and gut compartments. Clinically, such urinary signatures may provide non-invasive biomarkers, and microbiome-directed intervention may represent a future therapeutic avenue. Longitudinal, mechanistic, and multi-omic studies, ideally incorporating paired urinary fractions and semen sampling, are warranted to establish causality and to translate these associations into clinical practice.

## Data Availability

The data are available by contacting the corresponding author.

## Abbreviations

AZS: Asthenozoospermia
ASV: Amplicon sequence variant
BMI: Body mass index
CASA: Computer-assisted sperm analysis
KEGG: Kyoto Encyclopedia of Genes and Genomes
LDA: Linear discriminant analysis
LEfSe: Linear discriminant analysis effect size
LPS: Lipopolysaccharide
PBS: Phosphate-buffered saline
PCoA: Principal coordinate analysis
PERMANOVA: Permutational multivariate analysis of variance
PICRUSt2: Phylogenetic Investigation of Communities by Reconstruction of Unobserved States 2
qPCR: Quantitative polymerase chain reaction
QIIME2: Quantitative Insights Into Microbial Ecology 2
ROS: Reactive oxygen species
SCFA: Short-chain fatty acid
TLR4: Toll-like receptor 4
UTI: Urinary tract infection
WHO: World Health Organization
